# Individual and Contextual Factors Associated with the Need for and Use of Dental Prostheses in Brazil: A Multilevel Analysis of SB Brasil 2023 Data

**DOI:** 10.1590/1980-549720260003.supl.1

**Published:** 2026-05-08

**Authors:** Lucas Xavier Bezerra de Menezes, Laura Maria de Almeida Martins, Maria Letícia Barbosa Raymundo, Rilary Rodrigues Feitosa, Niely Enetice de Sousa Catão, Armando Cabral de Lira, Rennis Oliveira da Silva, Rafael Aiello Bomfim, Yuri Wanderley Cavalcanti

**Affiliations:** I Universidade Federal da Paraíba, Graduate Program in Dentistry - João Pessoa (PB), Brazil.; II Universidade Federal de Pernambuco, Graduate Program in Dentistry - Recife (PE), Brazil.; III Universidade Federal do Mato Grosso do Sul, Department of Preventive Dentistry - Campo Grande (MS), Brazil.; IV Universidade Federal da Paraíba, Department of Clinical and Social Dentistry - João Pessoa (PB), Brazil.

**Keywords:** Public health, Dentistry, Dental prosthesis, Socioeconomic factors

## Abstract

**Objective:**

To evaluate how individual and contextual socioeconomic variables influence the prevalence of denture use and need among Brazilian older adults, considering the National Oral Health Survey (SB Brasil 2023) and the expansion of primary and specialized care.

**Methods:**

This is a quantitative, analytical, cross-sectional study with 9,745 older adults (65-74 years) from SB Brasil 2023, whose outcomes are “need” and “use” of dentures. Associations are analyzed using multilevel logistic regression with random intercept by municipality, reporting odds ratios and 95% confidence intervals with a robust estimator.

**Results:**

The model for denture need indicated higher odds among self-declared Black older adults, users of public services, and residents of municipalities with a higher proportion of Black people (p<0.05). For denture use, a higher probability was observed among women, people with per-capita income between the poverty line and two minimum wages, and schooling of 5-8 years (p<0.05). Access through the public service was associated with a lower probability of use (p<0.05).

**Conclusion:**

Denture use and need among older adults are marked by inequities by skin color, gender, income, and schooling. The availability of public dental services showed no significant effect, and strategies are needed that prioritize vulnerable groups and increase the efficiency of dental care in the Unified Health System.

## INTRODUCTION

The National Oral Health Policy (Política Nacional de Saúde Bucal - PNSB), launched in 2004, was grounded in the principles of universality, equity, and comprehensiveness that guide the Brazilian Unified Health System (Sistema Único de Saúde - SUS)^1^. Over 20 years of implementation, the PNSB has sought to ensure actions aimed at the promotion, prevention, and restoration of oral health for the Brazilian population^2^. Among other initiatives, PNSB contributed to the expansion of dental care within Primary Health Care through the establishment of Oral Health Teams^3^, as well as to the implementation of specialized dental services through Dental Specialty Centers (DSCs) and Regional Dental Prosthesis Laboratories (RDPL)^4^ ^,^ ^5^ ^,^ ^6^.

However, despite the advances achieved by the PNSB, Brazilian older adults continue to bear a high burden of oral diseases, with tooth loss and the need for prosthetic rehabilitation representing important markers of accumulated inequities throughout the life course^7^ ^,^ ^8^. Recent data from the 2023 National Oral Health Survey (SB Brasil 2023) indicate that caries experience and the prevalence of edentulism among Brazilian older adults have significantly declined over the past 20 years^9^. Nevertheless, inequities associated with skin color and educational attainment persist, resulting in disparities in the oral health conditions of this population^9^.

Between 2010 and 2023, edentulism among Brazilian older adults aged 65 to 74 years decreased from 53.4 to 36.48%^9^. Data from the 2010 National Oral Health Survey indicate that the prevalence of denture use was 78.2%, whereas the prevalence of denture need was 68.7%^10^. In comparison, data from 2023 show that 70.9% of individuals use dentures and 75.2% require dentures^11^. This scenario suggests that only limited changes occurred between 2010 and 2023, despite the expansion of oral health services within SUS^5^ ^,^ ^6^.

Most studies addressing the oral health conditions of older adults focus primarily on caries experience, tooth loss, edentulism, and difficulties in accessing health care^9^ ^,^ ^12^ ^,^ ^13^ ^,^ ^14^. However, relatively few investigations have examined the factors associated with the use of and need for dental prostheses as a strategy to improve access to care and to contribute to the restoration of oral function and quality of life in this population. Rehabilitation with dental prostheses, therefore, not only affects quality of life but also reflects the organization of public services and the capacity of the health system to respond equitably to the population’s needs^15^ ^,^ ^16^.

Previous studies have demonstrated that the use of and need for dental prostheses among older adults are associated with individual socioeconomic variables (gender, skin color, income, and educational attainment) and contextual variables (Human Development Index - HDI, type of municipality)^10^ ^,^ ^15^ ^,^ ^17^ ^,^ ^18^. In addition, self-perceived need for treatment and use of dental services have been identified as significant factors^10^ ^,^ ^17^. However, studies examining the use of and need for dental prostheses in older adults remain limited and have not addressed the contribution of the availability of oral health services within municipalities as a potential modulating factor of this condition. Furthermore, there is a scarcity of national investigations that simultaneously assess individual and contextual variables using multilevel models in the context of dental prosthesis use and need.

It is important to recognize that the need for prostheses does not refer solely to tooth loss, but rather to an individual’s need for dental treatment, which may include the replacement of an existing prosthesis. With respect to prosthesis use, this parameter may serve as an indicator of access to dental treatment. Thus, these two parameters may be used to assess both treatment need and individuals’ access to this type of rehabilitation. Inequalities shaped by social determinants should inform the organization of the health care network in order to promote greater equity.

Considering the expansion of dental care in primary and specialized services within SUS, as well as the potential influence of contextual variables across Brazilian municipalities, this study aimed to evaluate the effect of individual and contextual socioeconomic factors on the prevalence of dental prosthesis use and need among Brazilian older adults.

## METHODS

A quantitative, analytical, cross-sectional study was conducted using data from 9,745 older adults (65-74 years) who participated in the SB Brasil 2023 survey. The survey was approved on July 3, 2021, by the Research Ethics Committee, in accordance with the ethical principles established by Resolution No. 466, of December 12, 2012, of the National Health Council, CAAE No. 34497120.6.3001.0008 and opinion No. 4.823.054. The Informed Consent and the Informed Assent Forms were applied^11^. The present study was conducted using microdata provided by the Ministry of Health, upon submission of a commitment agreement by the researchers.

SB Brasil 2023 is a household-based survey that included the 27 Federative Units (FUs), encompassing the state capitals and municipalities in the interior of each FU (n = 26), resulting in 53 geographic domains^11^ ^,^ ^19^. Census tracts were randomly selected, followed by households, using a probabilistic cluster sampling technique^11^. A structured questionnaire addressing the demographic and socioeconomic characteristics of participants and their families was administered, and oral examinations were conducted under natural light using a flat mouth mirror and a World Health Organization (WHO) probe. The field teams consisted of one examiner (dentist) and two recorders. All team members completed online theoretical and practical training, and examiners additionally underwent calibration, with a minimum acceptable Kappa coefficient of 0.61^11^ ^,^ ^19^.

For the present study, two outcomes were considered: “need for prosthesis” and “use of prosthesis.” The variable “need for prosthesis” was categorized as “needs prosthesis” or “does not need prosthesis,” whereas “use of prosthesis” was categorized as “uses prosthesis” or “does not use prosthesis.” Both outcomes were derived from variables available in the complete and weighted SB Brasil 2023 database.

The individual predictor variables were as follows: gender (female or male), self-reported skin color (White, Black, or Brown), per capita family income in minimum wages (MW) (below the poverty line, at the poverty line up to 1 MW, 1 to 2 MW, or >2 MW), years of schooling (no formal education, 1 to 4 years, 5 to 8 years, or ≥9 years), and type of service where the last dental consultation was performed (public or private). Skin color was self-reported by participants according to the criteria adopted by the Brazilian Institute of Geography and Statistics (Instituto Brasileiro de Geografia e Estatística - IBGE). Asian and Indigenous individuals were excluded from the analysis because they represented less than 3% of the sample. Per capita family income was calculated as the ratio between the reported total family income and the number of household residents. The minimum wage considered for the study corresponded to the year in which the survey was conducted (R$ 1,320). The poverty line was defined as R$ 660, corresponding to 50% of the MW in effect during the data collection period (R$ 1,320) - a criterion widely used in national studies as an operational estimate of economic vulnerability.

The contextual predictor variables were as follows: proportion of Black individuals (up to 60% or >60%), oral health coverage in Primary Health Care (up to 79% or ≥80%), presence of DSCs (yes or no), and presence of RDPL (yes or no). Data on the proportion of Black individuals were obtained from the 2022 IBGE Census, based on the combined frequency of Black and Brown individuals within the municipal population. The remaining contextual variables were derived from publicly available reports on the e-Gestor APS portal, corresponding to the average estimates for the year 2023, coinciding with the data collection period of SB Brasil 2023.

Data were analyzed using Stata^®^ software, version 19 (StataCorp., College Station, United States). Weighted estimates were calculated, taking into account the complex sampling design^20^. Associations between the outcome and independent variables were assessed using multilevel logistic regression models with random intercepts at the municipal level. Estimates were obtained using the logit link function, and odds ratios (OR) with respective 95% confidence intervals (95%CI) were calculated using robust variance estimators.

Null models were generated to assess model fit and the parameters of the random intercept. Model 1 (unadjusted) included only individual-level variables (gender, skin color, income, education, and location of the last dental visit). Model 2 (final) included both individual and contextual variables. Individuals with missing data for any of the individual variables of interest were excluded from the regression analyses. The fit of the null model was compared with that of the adjusted models using the Akaike Information Criterion (AIC) and the Bayesian Information Criterion (BIC), with lower values indicating better model fit. The municipal-level effect was assessed using the Intraclass Correlation Coefficient (ICC). A reduction in AIC, BIC, and ICC values was observed in the final model, indicating improved model fit and decreased variability attributable to municipalities. Variables with p<0.05 were considered statistically significant.

## Data availability statement:

The entire dataset supporting the results of this study is available upon request to the corresponding author. The dataset is not publicly available due to database integration performed by the authors.

## RESULTS

Table 1 presents the absolute and weighted frequencies of the sample according to the outcome variables, as well as the individual and contextual predictors. Of the total older adults included in the study (n=9,754), 75.2% required a prosthesis, and 70.9% used some type of prosthesis. The sample was predominantly composed of females, White individuals, and participants with per capita family income between the poverty line and 1 MW. Most had more than eight years of schooling and a lower proportion reported use of dental services within the public sector. Regarding contextual characteristics, participants predominantly resided in municipalities with a lower proportion of Black individuals, lower oral health coverage, and with the presence of DSCs and RDPL.

**Table 1.** Descriptive characteristics of older adults who participated in the 2023 National Oral Health Survey (n=9,745), considering outcome, individual, and contextual variables. Values represent absolute frequency and weighted estimate (% (95%CI)). For contextual variables, absolute frequency by municipality is presented.

Characteristics	Categories	Absolute frequency	Weighted estimate (% (95%CI))
Needs dental prosthesis	No	2,305	24.8 (21.9-27.8)
	Yes	7,391	75.2 (72.2-78.1)
Uses dental prosthesis	No	2,694	29.1 (26.4-31.9)
	Yes	7,002	70.9 (68.1-73.6)
Gender	Female	3,710	60.4 (58.2-62.7)
	Male	6,034	39.6 (37.3-41.9)
Skin color	White	3,601	51.7 (47.8-55.6)
	Black	1,353	12.4 (10.7-14.2)
	Brown	4,465	35.9 (31.9-40.1)
Per capita household income	Up to the poverty line	2,564	27.2 (23.7-31.1)
	Poverty line to 1 MW	2,830	42.5 (38.7-46.4)
	1 to 2 MW	1,179	22.6 (17.9-28.2)
	More than 2 MW	611	7.7 (5.9-9.9)
Education (years of schooling)	No formal education	1,240	11.5 (9.8-13.5)
	1 to 4	2,099	27.9 (24-32.1)
	5 to 8	2,484	26.2 (23.7-29)
	More than 8	3,687	34.3 (7.4-11.3)
Type of service (last visit)	Non-public	4,997	62.5 (58.3-66.4)
	Public	3,722	37.5 (33.6-41.7)
Proportion of Black population (%)	Up to 60	4,494 (170 municipalities)	65.8 (61.4-70)
	>60	5,251 (198 municipalities)	34.2 (30-38.6)
Oral health coverage (%)	Up to 80	7,300 (172 municipalities)	72.3 (67-77)
	>80	2,445 (196 municipalities)	27.7 (22.9-33)
Presence of DSC	No	1,189 (164 municipalities)	26.9 (10.8-33.9)
	Yes	8,556 (204 municipalities)	73.1 (66.1-79.2)
Presence of RDPL	No	1,750 (116 municipalities)	24.2 (18.4-31.1)
	Yes	7,995 (252 municipalities)	75.8 (68.9-81.6)

95%CI: 95% confidence interval; MW: minimum wage(s); DSC: Dental Specialty Center; RDPL: Regional Dental Prosthesis Laboratory.

The adjusted logistic regression model estimating factors associated with the need for dental prostheses (Table 2) included 6,011 individuals with complete data. The analysis indicated that self-declared Black older adults, those who accessed public dental services, and those residing in municipalities with a higher proportion of Black individuals were more likely to require prostheses (p<0.05). In contrast, women, individuals with higher income, and those with more years of schooling were less likely to require prostheses (p<0.05). No significant effects were observed for contextual variables related to the availability of dental services within SUS (including oral health coverage, presence of DSCs, and presence of RDPL) (p>0.05). Approximately 18% of the variance in the adjusted model was attributable to differences between municipalities.

**Table 2.** Multilevel logistic regression to estimate factors associated with the need for dental prosthesis among older adults (Brasil, 2023).

Characteristics	Model 1		Model 2	
	OR	95%CI	OR	95%CI
Gender (Male)	ref.		ref.	
Female	0.85	0.75-0.97	0.84	0.73-0.96
Skin color (White)	ref.		ref.	
Black	1.34	1.10-1.63	1.38	1.14-1.67
Brown	1.15	0.98-1.35	1.11	0.93-1.32
Income (Up to the poverty line)	ref.		ref.	
Poverty line to 1 MW	0.86	0.74-1.01	0.91	0.64-1.29
1 MW to 2 MW	0.52	0.41-0.65	0.7	0.52-0.94
More than 2 MW	0.28	0.21-0.37	0.34	0.24-0.49
Education (No formal education)	ref.		ref.	
1 to 4 years	0.86	0.65-1.13	0.93	0.70-1.24
5 to 8 years	0.70	0.51-0.97	0.75	0.54-1.04
More than 8 years	0.66	0.51-0.86	0.69	0.53-0.91
Place of last visit (Non-public)	ref.		ref.	
Public	1.84	1.56-2.16	1.86	1.59-2.17
Proportion of Black population (Up to 60%)				
High (>60%)			1.41	1.02-1.95
Oral health coverage (Up to 80%)				
High (>80%)			0.94	0.69-1.27
Presence of DSC (No)				
Yes			1.03	0.70-1.50
Presence of RDPL (No)				
Yes			0.93	0.63-1.37

OR: odds ratio; 95%CI: 95% confidence interval; Null model parameters: ICC=0.22; AIC=10,252.3; BIC=10,266.7. Adjusted model parameters: ICC=0.18; AIC=6,141.6; BIC=6,248.8.

The adjusted logistic regression model estimating factors associated with the use of dental prostheses (Table 3) included 6,005 cases with complete data. The analysis demonstrated that older women, individuals with per capita family income between the poverty line and 2 MWs, and those with 5 to 8 years of schooling were more likely to use dental prostheses (p<0.05). Older adults who accessed public dental services were less likely to use dental prostheses (p<0.05). No statistically significant effects were observed for the contextual variables (p>0.05). Approximately 6% of the variance in the adjusted model was attributable to differences between municipalities.

**Table 3.** Multilevel logistic regression to estimate factors associated with the use of dental prosthesis among older adults (Brasil, 2023).

Characteristics	Model 1		Model 2	
	OR	95%CI	OR	95%CI
Gender (Male)	ref.		ref.	
Female	1.79	1.54-2.09	1.78	1.52-2.09
Skin color (White)	ref.		ref.	
Black	0.88	0.75-1.04	0.93	0.78-1.10
Brown	0.99	0.86-1.13	1.02	0.88-1.18
Income (Up to the poverty line)	ref.		ref.	
Poverty line to 1 MW	1.31	1.14-1.50	1.44	1.14-1.82
1 MW to 2 MW	1.24	0.99-1.55	1.74	1.37-2.22
More than 2 MW	0.69	0.53-0.91	1.17	0.88-1.56
Education (No formal education)	ref.		ref.	
1 to 4 years	1.23	1.01-1.49	1.21	0.99-1.48
5 to 8 years	1.37	1.09-1.71	1.36	1.08-1.72
More than 8 years	0.97	0.76-1.24	0.96	0.75-1.24
Place of last visit (Non-public)	ref.		ref.	
Public	0.56	0.48-0.65	0.57	0.50-0.66
Proportion of Black population (Up to 60%)				
High (>60%)			0.86	0.68-1.09
Oral health coverage (Up to 80%)				
High (>80%)			1.21	0.98-1.50
Presence of DSC (No)				
Yes			0.79	0.59-1.06
Presence of RDPL (No)				
Yes			1.05	0.79-1.39

OR: odds ratio; 95%CI: 95% confidence interval; Null model parameters: ICC=0.09; AIC=11,352.8; BIC=11,367.2. Adjusted model parameters: ICC=0.06; AIC=6,801.1; BIC=6,908.3.

## DISCUSSION

The findings of this study indicate that the need for dental prostheses among Brazilian older adults remains high and is associated with socioeconomic inequalities related to skin color, gender, income, education, and access to dental services. Overall, no substantial changes were observed compared with the scenarios reported in 2003 and 2010^10^ ^,^ ^15^ ^,^ ^17^ ^,^ ^18^. However, significant variation was identified among Brazilian municipalities, and the availability of public oral health services was not associated with the need for prostheses among older adults.

These findings indicate that the need for dental prostheses among Brazilian older adults is marked by socioeconomic disparities, including differences related to skin color. A previous study analyzing the provision of dental prostheses in Brazil demonstrated that municipalities with a higher proportion of Black individuals exhibit greater inequities in the supply of dental prostheses within SUS^21^. The present results corroborate these findings, showing that Black older adults and those residing in municipalities with a higher proportion of Black individuals are more likely to require prostheses. In contrast, these factors were not associated with prosthesis use.

In the Brazilian context, the variable “skin color” does not represent a biological marker, but rather a social marker that reflects structural inequalities resulting from historical processes of racialization and structural racism. Studies have demonstrated that Black populations experience reduced access to prosthetic rehabilitation and a higher prevalence of edentulism, even after adjustment for income and education^9^ ^,^ ^21^. The present findings reinforce that the effects of race/skin color extend beyond the individual level and manifest contextually, as municipalities with a higher proportion of Black residents exhibit a greater prevalence of prosthesis need. This pattern suggests an intersectional mechanism in which race and socioeconomic status interact, intensifying vulnerabilities accumulated over the life course. In this study, the variable “race/skin color” was not used as a proxy for socioeconomic status but was included in the statistical model as a marker of social inequity. The results indicate that Black individuals had higher odds of denture need, although no differences were observed regarding prosthesis use.

The greater availability of dental prostheses in municipalities with a higher number of Oral Health Teams in Primary Health Care and DSCs, as previously reported^21^, contrasts with the findings of the present study, which did not demonstrate significant effects of oral health coverage in PHC or of the presence of DSCs and RDPLs at the municipal level. In this study, the high proportion of individuals residing in municipalities with available public oral health services may have influenced the results. Additionally, it is possible that older adults do not seek public dental services for the fabrication and placement of dental prostheses.

A recent study demonstrated that the expansion of Oral Health Teams in Primary Health Care within SUS occurred predominantly in less populated municipalities with lower GDP per capita in the Northeast region^3^. These findings suggest that PNSB prioritized municipalities with greater social vulnerability; this indicator is currently used to support the implementation of Oral Health Teams. According to the results of the present study, no significant effect of greater availability of dental services within SUS was observed on the need for or use of dental prostheses among Brazilian older adults. It was hypothesized that municipalities with the presence of DSCs and RDPLs might exhibit lower prosthesis need and higher prosthesis use; however, this effect was not identified.

Therefore, it is suggested that oral health services prioritize individuals experiencing greater socioeconomic vulnerability. Currently, there are no standardized protocols or guidelines to direct the screening of users with dental needs for triage and care within SUS. The findings of the present study may contribute to the development of algorithms and technological tools to support this process. Furthermore, the expansion of dental services within SUS should consider territories with higher levels of population vulnerability, particularly with respect to income, education, and skin color.

With respect to the use of dental prostheses, individual socioeconomic variables also demonstrated a significant association with the outcome, consistent with previous findings in the literature^10^ ^,^ ^15^ ^,^ ^17^ ^,^ ^18^. Older adults with lower income and lower educational attainment (reference categories) exhibited a lower frequency of prosthesis use. These groups also presented the greatest need for prostheses, indicating that this condition remains highly prevalent among Brazilian older adults.

It is important to emphasize that the use of dental prostheses is not equivalent to the mere absence of teeth. Individuals with tooth loss may or may not use prostheses and may or may not require rehabilitative treatment. Thus, prosthesis use indicates that the individual has had access to rehabilitative care. Furthermore, national studies^10^ ^,^ ^15^ ^,^ ^18^ have demonstrated that the determinants of prosthesis use and need are partially distinct from those associated with tooth loss.

The absence of differences in prosthesis use across the extreme categories of income and education may be explained by the lower prevalence of edentulism among older adults with higher income and higher educational attainment^9^, whereas those with lower income and lower education (reference categories) may encounter barriers to accessing dental services^13^ ^,^ ^14^, particularly within the public sector, which was also significantly associated with a lower frequency of prosthesis use.

In the present study, older adults who used public dental services exhibited a lower frequency of both need for and use of dental prostheses. Additionally, the availability of public dental services did not influence the frequency of prosthesis use or need. These findings may be explained by evidence indicating low utilization of dental services among older adults^13^ ^,^ ^14^. It is noteworthy, however, that both dental service utilization and prosthesis use and need are marked by socioeconomic inequities. Accordingly, measures are required to reduce these disparities and to better direct oral health actions within SUS toward the populations with the greatest needs.

This study has limitations related to the non-response rate, categorization of the variables analyzed, participant characteristics, and the imbalance inherent to the complex sampling design. With respect to non-response - approximately 27% for income data - although these individuals were excluded from the analyses, the statistical models were considered adequately adjusted and robust for inference.

In the present study, the type of dental prosthesis was not considered in the analysis of prosthesis use and need among Brazilian older adults. From an organizational perspective, with regard to service resource allocation, the types of dental prostheses produced are not differentiated; rather, only the total quantity is recorded. RDPLs are included within overall production ranges (without distinction by prosthesis type). Therefore, the analysis of factors associated with prosthesis use and need among Brazilian older adults is not compromised.

The analyses conducted in this study included all older adults, regardless of enrollment in private dental insurance plans. This approach was adopted to provide a comprehensive assessment of the Brazilian older adult population as a whole. Given that SUS is a universal health system, individuals covered by private plans are also encompassed within the scope of public health services.

The dichotomizations adopted in this study were based on contextual variables derived from indicators provided by the Ministry of Health, such as oral health coverage and the presence of DSCs, which are used to guide public policies. With respect to individual variables, multiple categories were adopted in order to allow the assessment of potential social gradients.

Although DSCs are not necessarily responsible for the provision of dental prostheses, these services reflect local management efforts to offer specialized care. In many municipalities, clinical procedures for prosthesis fabrication are integrated into DSCs, although the actual production and delivery of prostheses depend on the structure of RDPLs. Moreover, the presence of DSCs may indicate greater organization of the oral health care network, improved qualification of care pathways, and an increased likelihood of referral to RDPLs. Previous studies have demonstrated that municipalities with a higher density of specialized services exhibit better overall performance of the oral health network. Accordingly, this variable was included as an indicator of installed capacity and network organization, which may directly or indirectly influence rehabilitation outcomes.

Furthermore, the greater sample weight assigned to individuals residing in capital cities may be associated with increased availability of DSCs and RDPL, as well as lower oral health coverage in PHC. It should also be considered that a substantial proportion of these individuals may not seek dental care within SUS. Therefore, the influence of the availability of public dental services should be interpreted with caution.

The results of this investigation allow us to conclude that individual socioeconomic variables significantly influence the need for and use of dental prostheses among aged adults. Among the contextual variables, only the higher proportion of Black people in the municipalities was associated with a greater need for dental prostheses, revealing that the municipal context can also influence the results and highlighting inequities possibly related to racism.

Considering that the prevalence of need for and use of dental prostheses among Brazilian older adults remained stable between 2010 and 2023, it is plausible that the expansion of dental services within SUS did not influence these estimates. These findings underscore the need to develop strategies and tools capable of modifying patterns of prosthesis use and need in the population. It is noteworthy that 49.6% of Brazilian adults (aged 35 to 44 years) neither use nor require any type of prosthesis^22^. Therefore, it is evident that PNSB must implement strategies to expand the availability of prostheses within SUS. In the absence of such measures, this scenario may remain unchanged among Brazilian older adults over the next 10 to 20 years.

In summary, the use of and need for dental prostheses among Brazilian older adults are marked by socioeconomic inequities related to gender, skin color, income, and education. Utilization of public oral health services was associated with greater prosthesis need and lower prosthesis use among older adults. At the contextual level, a higher proportion of Black individuals within municipalities was associated with greater prosthesis need, whereas the availability of dental services within SUS did not demonstrate a significant effect.

In light of these findings, it is necessary to adopt strategies aimed at prioritizing the provision of dental prostheses to individuals in the most vulnerable conditions, implementing approaches that enhance the efficiency of dental care delivered by oral health services within SUS.
