# Supplementary material for: Individual and Contextual Factors Associated with the Need for and Use of Dental Prostheses in Brazil: A Multilevel Analysis of SB Brasil 2023 Data
Source: Rev Bras Epidemiol. 2026 May 8;29(Suppl 1):e260003.supl.1. doi: 10.1590/1980-549720260003.supl.1 (PMC13181637; doi:10.1590/1980-549720260003.supl.1)
Supplement: Translation

# Translation: Fatores individuais e contextuais associados à necessidade e ao uso de próteses dentárias no Brasil: uma análise multinível dos dados da Pesquisa Nacional de Saúde Bucal 2023

**DOI:** 10.1590/1980-549720260003.supl.1.2

**Authors:** Lucas Xavier Bezerra de Menezes, Laura Maria de Almeida Martins, Maria Letícia Barbosa Raymundo, Rilary Rodrigues Feitosa, Niely Enetice de Sousa Catão, Armando Cabral de Lira, Rennis Oliveira da Silva, Rafael Aiello Bomfim, Yuri Wanderley Cavalcanti

**Affiliations:** I Universidade Federal da Paraíba, Programa de Pós-Graduação em Odontologia - João Pessoa (PB), Brasil.; II Universidade Federal de Pernambuco, Programa de Pós-Graduação em Saúde da Criança e do Adolescente - Recife (PE), Brasil.; III Universidade Federal do Mato Grosso do Sul, Departamento de Odontologia Preventiva - Campo Grande (MS), Brasil.; IV Universidade Federal da Paraíba, Departamento de Clínica e Odontologia Social - João Pessoa (PB), Brasil.

**Keywords:** Saúde pública, Odontologia, Prótese dentária, Fatores socioeconômicos

## Abstract

**Objetivo:**

Avaliar como variáveis socioeconômicas individuais e contextuais influenciam a prevalência de uso e necessidade de prótese em idosos brasileiros, considerando a Pesquisa Nacional de Saúde Bucal 2023 (SB Brasil 2023) e a expansão da atenção primária e especializada.

**Métodos:**

Trata-se de um estudo quantitativo, analítico, transversal, com 9.745 idosos (65-74 anos) do SB Brasil 2023, que tem como desfechos: “necessidade” e “uso” de prótese. São analisadas associações por regressão logística multinível com intercepto aleatório por município, reportando a razão de chances e o intervalo de confiança de 95% com estimador robusto.

**Resultados:**

O modelo para necessidade de prótese indicou maior chance para idosos autodeclarados pretos, usuários do serviço público e residentes em municípios com maior proporção de negros (p<0,05). Para o uso de prótese, observou-se maior probabilidade entre mulheres, pessoas com renda per capita entre a linha da pobreza e dois salários mínimos e escolaridade de 5-8 anos (p<0,05). O acesso pelo serviço público associou-se a menor probabilidade de uso (p<0,05).

**Conclusão:**

O uso e a necessidade de próteses em idosos são marcados por iniquidades segundo cor da pele, sexo, renda e escolaridade. A disponibilidade de serviços odontológicos públicos não mostrou efeito significativo, sendo necessárias estratégias que priorizem grupos vulneráveis e elevem a eficiência da atenção odontológica no Sistema Único de Saúde (SUS).

## INTRODUÇÃO

A Política Nacional de Saúde Bucal (PNSB), lançada em 2004, foi fundamentada nos princípios da universalidade, equidade e integralidade que orientam o Sistema Único de Saúde (SUS) no Brasil^1^. Ao longo de 20 anos de implementação, a PNSB teve como objetivo garantir ações de promoção, prevenção e recuperação da saúde bucal da população brasileira^2^. Entre outras ações, a PNSB contribuiu para a expansão da assistência odontológica na Atenção Primária à Saúde, por meio das Equipes de Saúde Bucal^3^, bem como para a implementação da atenção odontológica especializada, por intermédio de Centros de Especialidades Odontológicas (CEOs) e Laboratórios Regionais de Próteses Dentárias (LRPD)^4^ ^,^ ^5^ ^,^ ^6^.

Entretanto, apesar dos avanços da PNSB, os idosos brasileiros acumulam uma carga elevada de doenças bucais, sendo a perda dentária e a necessidade de reabilitação protética marcadores importantes de iniquidades acumuladas ao longo do curso de vida^7^ ^,^ ^8^. Dados recentes da Pesquisa Nacional de Saúde Bucal 2023 (SB Brasil 2023) revelam que a experiência de cárie e a prevalência de edentulismo em idosos brasileiros reduziram significativamente nos últimos 20 anos^9^. Entretanto, iniquidades marcadas pela cor da pele e pela escolaridade ainda continuam a produzir diferenças nas condições de saúde bucal desses indivíduos^9^.

Entre 2010 e 2023, o edentulismo em idosos brasileiros de 65 a 74 anos reduziu de 53,4 para 36,48%^9^. Dados do inquérito de saúde bucal de 2010 revelam que a prevalência do uso de prótese foi de 78,2%, enquanto a de necessidade de prótese foi de 68,7%^10^. Entretanto, ao estabelecer um paralelo com 2023, verifica-se que 70,9% usam e 75,2% necessitam de prótese^11^. Esse cenário revela que poucas mudanças ocorreram entre 2010 e 2023, apesar da expansão dos serviços de saúde bucal no SUS^5^ ^,^ ^6^.

A maior parte dos estudos sobre as condições de saúde bucal de idosos foca suas análises em experiência de cárie, perda dentária, edentulismo e dificuldades de acesso à saúde^9^ ^,^ ^12^ ^,^ ^13^ ^,^ ^14^. Entretanto, poucos estudos se debruçaram sobre os fatores associados a uso e necessidade de prótese dentária como uma estratégia de melhorar o acesso e contribuir para restabelecimento da função oral e qualidade de vida da população idosa. A reabilitação por meio de próteses dentárias, portanto, não apenas impacta a qualidade de vida, mas também reflete a organização dos serviços públicos e a capacidade do sistema de saúde em responder de forma equitativa às necessidades da população^15^ ^,^ ^16^.

Estudos prévios verificaram que o uso e a necessidade de próteses dentárias em idosos foram associados a variáveis socioeconômicas individuais (sexo, cor da pele, renda e escolaridade) e contextuais (Índice de Desenvolvimento Humano - IDH, tipo de município)^10^ ^,^ ^15^ ^,^ ^17^ ^,^ ^18^. Além disso, a autopercepção de necessidade de tratamento e uso de serviços odontológicos foi reportada como fator significativo^10^ ^,^ ^17^. Entretanto, os estudos sobre uso e necessidade de prótese dentária em idosos são escassos e não abordam a contribuição da disponibilidade dos serviços de saúde bucal nos municípios como possível fator modulador dessa condição, bem como há uma escassez de investigações nacionais que avaliem simultaneamente variáveis individuais e contextuais em modelos multiníveis, sob o contexto do uso e da necessidade de prótese dentária.

É relevante compreender que a necessidade de prótese não se refere apenas à ausência de dentes, mas sim a uma necessidade de tratamento odontológico do indivíduo, que pode incluir a substituição de uma eventual prótese já instalada. Em relação ao uso de prótese, este pode constituir um indicador de acesso ao tratamento odontológico. Dessa forma, esses dois parâmetros podem medir a necessidade de tratamento e o acesso dos indivíduos a este tipo de reabilitação. Desigualdades marcadas por fatores sociais devem orientar a rede de atenção à saúde de maneira mais equânime.

Considerando a expansão da assistência odontológica na atenção primária e especializada do SUS, e o possível efeito de variáveis contextuais nos municípios brasileiros, este trabalho tem por objetivo avaliar a influência de variáveis socioeconômicas individuais e contextuais na prevalência de uso e necessidade de prótese em idosos brasileiros.

## MÉTODOS

Realizou-se um estudo quantitativo, analítico e de corte transversal, que utilizou dados de 9.745 indivíduos idosos (65-74 anos) que participaram da SB Brasil 2023. O referido inquérito foi aprovado em 3 de julho de 2021 pelo Comitê de Ética em Pesquisa, seguindo os aspectos éticos da Resolução n.º 466, de 12 de dezembro de 2012, do Conselho Nacional de Saúde, sob CAAE 34497120.6.3001.0008 e parecer 4.823.054; assim como foram utilizados o Termo de Consentimento Livre e Esclarecido e o Termo de Assentimento Livre e Esclarecido^11^. Este estudo foi realizado com base nos microdados disponibilizados pelo Ministério da Saúde, mediante apresentação de termo de compromisso dos pesquisadores.

O SB Brasil 2023 é um inquérito de base domiciliar, que teve como domínios de estudo as Unidades Federativas (UF) (n=27), as capitais e os municípios do interior de cada UF (n=26), resultando em 53 domínios geográficos^11^ ^,^ ^19^. Foram sorteados os setores censitários e, em seguida, os domicílios por meio da técnica de amostragem probabilística por conglomerados^11^. Um questionário direcionado aos aspectos demográficos e socioeconômicos dos participantes e de suas famílias foi utilizado, e os exames bucais foram realizados sob luz natural, com espelho bucal plano e sonda OMS. As equipes foram compostas por um examinador (cirurgião-dentista), um anotador e um arrolador. Todos se submeteram a um treinamento teórico-prático online e os examinadores, adicionalmente, à calibração, cujo coeficiente Kappa mínimo aceitável foi de 0,61^11^ ^,^ ^19^.

Para este estudo, foram considerados dois desfechos: “necessidade de prótese” e “uso de prótese”. A variável “necessidade de prótese” foi categorizada em “necessita de prótese” ou “não necessita de prótese”, enquanto “uso de prótese” foi categorizada em “usa prótese” ou “não usa prótese”, ambas provenientes das variáveis do banco de dados completo e ponderado do SB Brasil 2023.

As variáveis preditoras individuais foram as seguintes: sexo (feminino ou masculino), cor da pele autodeclarada (branca, preta ou parda), renda familiar per capita em Salários Mínimos (SM) (abaixo da linha da pobreza, linha da pobreza até 1 SM, 1 a 2 SM ou >2 SM), escolaridade em anos de estudo (não estudou, 1 a 4 anos, 5 a 8 anos ou ≥9 anos) e tipo de serviço onde realizou a última consulta (público ou não público). A cor da pele foi questionada aos participantes do estudo, considerando o critério adotado pelo Instituto Brasileiro de Geografia e Estatística (IBGE). Asiáticos e indígenas foram excluídos na análise, por representarem menos de 3% da amostra. A renda familiar per capita foi calculada de acordo com a razão entre a renda familiar informada e o número de moradores da residência. O valor do salário mínimo considerado para o estudo foi o relativo ao ano de realização da pesquisa (R$ 1.320). O limite da linha da pobreza considerado foi o valor de R$ 660, que corresponde a 50% do salário mínimo vigente no período da coleta (R$ 1.320) - critério amplamente utilizado em estudos nacionais como estimador operacional de vulnerabilidade econômica.

As variáveis preditoras contextuais foram as seguintes: proporção de negros (até 60% ou >60%), cobertura de saúde bucal na Atenção Primária a Saúde (até 79% ou ≥80%), presença de CEOs (sim ou não) e presença de LRPD (sim ou não). Os dados de proporção de negros foram extraídos do Censo 2022 do IBGE, sendo considerada a frequência de pretos e pardos na população municipal. Os demais dados referentes às variáveis contextuais foram extraídos dos relatórios públicos do portal e-gestorAPS, correspondendo à média das estimativas para o ano 2023, coincidente com a coleta de dados do SB Brasil 2023.

Os dados foram analisados utilizando-se o software Stata^®^, versão 19 (StataCorp., College Station, Estados Unidos). Estimativas ponderadas foram obtidas, considerando o delineamento complexo do estudo^20^. As associações entre as variáveis de desfecho e as variáveis independentes foram verificadas por meio de modelos de regressão logística multinível com intercepto aleatório por município. As estimativas foram obtidas utilizando-se a função de ligação logit, sendo calculadas medidas de Razão de Chance (OR) e Intervalo de Confiança 95% (IC95%) por estimador robusto.

Modelos nulos foram gerados para verificar o ajuste e os parâmetros do intercepto aleatório. O modelo 1 (não ajustado) incluiu apenas variáveis individuais (sexo, cor da pele, renda, escolaridade e local da última consulta odontológica). O modelo 2 (final) incluiu as variáveis individuais e contextuais. Indivíduos que não apresentaram todas as variáveis individuais de interesse foram excluídos do modelo de regressão. O ajuste do modelo nulo foi comparado ao modelo ajustado por meio dos critérios de informação AIC (Critério de Informação de Akaike) e BIC (Critério de Informação Bayesiano), sendo considerado melhor ajuste aquele que apresentou menores valores dessas medidas. O efeito dos municípios foi avaliado pelo Coeficiente de Correlação Intraclasse (ICC). Observou-se redução dos valores de AIC, BIC e do ICC no modelo final, indicando melhora no ajuste e redução da variabilidade atribuída aos municípios. Variáveis com p<0,05 foram consideradas estatisticamente significativas.

## Declaração de disponibilidade de dados:

Todo o conjunto de dados que dá suporte aos resultados deste estudo está disponível mediante solicitação ao autor correspondente. O conjunto de dados não está publicamente disponível devido à integração de bancos de dados realizada pelos autores.

## RESULTADOS

A Tabela 1 apresenta as frequências absolutas e ponderadas da amostra, considerando as variáveis de desfecho, assim como variáveis individuais e contextuais. Do total de idosos incluídos no estudo (n=9.754), 75,2% necessitam de prótese, e 70,9% usam algum tipo de prótese. Verifica-se que a amostra foi composta, em sua maioria, por idosos do sexo feminino, brancas, com renda familiar per capita entre a linha de pobreza e 1 salário mínimo, com escolaridade de mais de 8 anos de estudo e com menor proporção de utilização de serviços odontológicos na rede pública. Em relação às variáveis contextuais, os participantes residiram em municípios com menor proporção de negros, menor cobertura de saúde bucal, e em municípios com a presença de CEO e LRPD.

**Tabela 1.** Características descritivas dos idosos que participaram da Pesquisa Nacional de Saúde Bucal 2023 (n=9.745), considerando variáveis de desfecho, individuais e contextuais. Valores representam frequência absoluta e estimativa ponderada (% (IC95%)). Para variáveis contextuais, foi apresentada frequência absoluta por municípios.

Variáveis	Categorias	Frequência absoluta	Estimativa ponderada (% (IC95%))
Necessita de prótese	Não	2.305	24,8 (21,9-27,8)
	Sim	7.391	75,2 (72,2-78,1)
Usa prótese	Não	2.694	29,1 (26,4-31,9)
	Sim	7.002	70,9 (68,1-73,6)
Sexo	Feminino	3.710	60,4 (58,2-62,7)
	Masculino	6.034	39,6 (37,3-41,9)
Cor da pele	Branca	3.601	51,7 (47,8-55,6)
	Preta	1.353	12,4 (10,7-14,2)
	Parda	4.465	35,9 (31,9-40,1)
Renda familiar per capita	Até a linha da pobreza	2.564	27,2 (23,7-31,1)
	Linha da pobreza a 1 SM	2.830	42,5 (38,7-46,4)
	1 a 2 SM	1.179	22,6 (17,9-28,2)
	Mais de 2 SM	611	7,7 (5,9-9,9)
Escolaridade (anos de estudo)	Não estudou	1.240	11,5 (9,8-13,5)
	1 a 4	2.099	27,9 (24-32,1)
	5 a 8	2.484	26,2 (23,7-29)
	Mais de 8	3.687	34,3 (7,4-11,3)
Tipo de serviço (última consulta)	Não público	4.997	62,5 (58,3-66,4)
	Público	3.722	37,5 (33,6-41,7)
Proporção de negros (%)	Até 60	4.494 (170 municípios)	65,8 (61,4-70)
	>60	5.251 (198 municípios)	34,2 (30-38,6)
Cobertura de saúde bucal (%)	Até 80	7.300 (172 municípios)	72,3 (67-77)
	>80	2.445 (196 municípios)	27,7 (22,9-33)
Presença de CEO	Não	1.189 (164 municípios)	26,9 (10,8-33,9)
	Sim	8.556 (204 municípios)	73,1 (66,1-79,2)
Presença de LRPD	Não	1.750 (116 municípios)	24,2 (18,4-31,1)
	Sim	7.995 (252 municípios)	75,8 (68,9-81,6)

IC95%: intervalo de confiança de 95%; SM: salário(s) mínimo(s); CEO: Centro de Especialidade Odontológica; LRPD: Laboratório Regional de Prótese Dentária.

O modelo de regressão logística ajustado para estimar os fatores associados à necessidade de prótese dentária (Tabela 2) incluiu a análise de 6.011 indivíduos com dados completos. A análise revelou que idosos autodeclarados pretos, que acessaram o serviço público e residem em municípios com maior proporção de negros possuem maior necessidade de prótese (p<0,05). Idosos do sexo feminino, com maior renda e mais anos de estudo possuem menor necessidade (p<0,05). Não foram observados efeitos das variáveis contextuais relacionadas à disponibilidade de serviços odontológicos no SUS (cobertura de saúde bucal, presença de CEO e presença de LRPD) (p>0,05). Observou-se que 18% da variação do modelo ajustado pode ser atribuída aos municípios.

**Tabela 2.** Regressão logística multinível para estimar os fatores associados à necessidade de prótese dentária em idosos (Brasil, 2023).

Variáveis	Modelo 1		Modelo 2	
	OR	IC95%	OR	IC95%
Sexo (Masculino)	ref.		ref.	
Feminino	0,85	0,75-0,97	0,84	0,73-0,96
Cor da pele (Branca)	ref.		ref.	
Preta	1,34	1,10-1,63	1,38	1,14-1,67
Parda	1,15	0,98-1,35	1,11	0,93-1,32
Renda (Até a linha da pobreza)	ref.		ref.	
Linha da pobreza até 1 SM	0,86	0,74-1,01	0,91	0,64-1,29
1 SM a 2 SM	0,52	0,41-0,65	0,7	0,52-0,94
Mais do que 2 SM	0,28	0,21-0,37	0,34	0,24-0,49
Escolaridade (Não estudou)	ref.		ref.	
1 a 4 anos	0,86	0,65-1,13	0,93	0,70-1,24
5 a 8 anos	0,70	0,51-0,97	0,75	0,54-1,04
Mais de 8 anos	0,66	0,51-0,86	0,69	0,53-0,91
Local da última consulta (Não público)	ref.		ref.	
Público	1,84	1,56-2,16	1,86	1,59-2,17
Proporção de negros (Até 60%)				
Alta (>60%)			1,41	1,02-1,95
Cobertura de saúde bucal (Até 80%)				
Alta (>80%)			0,94	0,69-1,27
Presença de CEO (Não)				
Sim			1,03	0,70-1,50
Presença de LRPD (Não)				
Sim			0,93	0,63-1,37

OR: razão de chances (odds ratio); IC95%: intervalo de confiança de 95%; Parâmetros do modelo nulo: ICC=0,22; AIC=10.252,3; BIC=10.266,7. Parâmetros do modelo ajustado: ICC=0,18; AIC=6.141,6; BIC=6.248,8.

O modelo de regressão logística ajustado para estimar os fatores associados ao uso de prótese dentária (Tabela 3) incluiu 6.005 casos com dados completos. A análise revelou que idosos do sexo feminino, com renda familiar per capita entre a linha da pobreza e 2 salários mínimos, e com escolaridade de 5 a 8 anos de estudo possuem maior probabilidade de uso de prótese dentária (p<0,05). Idosos que acessaram o serviço público tiveram menor probabilidade de usar próteses dentárias (p<0,05). Não foram observados efeitos significativos de variáveis contextuais (p>0,05). Os municípios contribuíram para uma variação de 6% no modelo ajustado.

**Tabela 3.** Regressão logística multinível para estimar os fatores associados ao uso de prótese dentária em idosos (Brasil, 2023).

Variáveis	Modelo 1		Modelo 2	
	OR	IC95%	OR	IC95%
Sexo (Masculino)	ref.		ref.	
Feminino	1,79	1,54-2,09	1,78	1,52-2,09
Cor da pele (Branca)	ref.		ref.	
Preta	0,88	0,75-1,04	0,93	0,78-1,10
Parda	0,99	0,86-1,13	1,02	0,88-1,18
Renda (Até a linha da pobreza)	ref.		ref.	
Linha da pobreza até 1 SM	1,31	1,14-1,50	1,44	1,14-1,82
1 SM a 2 SM	1,24	0,99-1,55	1,74	1,37-2,22
Mais do que 2 SM	0,69	0,53-0,91	1,17	0,88-1,56
Escolaridade (Não estudou)	ref.		ref.	
1 a 4 anos	1,23	1,01-1,49	1,21	0,99-1,48
5 a 8 anos	1,37	1,09-1,71	1,36	1,08-1,72
Mais de 8 anos	0,97	0,76-1,24	0,96	0,75-1,24
Local da última consulta (Não público)	ref.		ref.	
Público	0,56	0,48-0,65	0,57	0,50-0,66
Proporção de negros (Até 60%)				
Alta (>60%)			0,86	0,68-1,09
Cobertura de saúde bucal (Até 80%)				
Alta (>80%)			1,21	0,98-1,50
Presença de CEO (Não)				
Sim			0,79	0,59-1,06
Presença de LRPD (Não)				
Sim			1,05	0,79-1,39

OR: razão de chances (odds ratio); IC95%: intervalo de confiança de 95%; Parâmetros do modelo nulo: ICC=0,09; AIC=11.352,8; BIC=11.367,2. Parâmetros do modelo ajustado: ICC=0,06; AIC=6.801,1; BIC=6.908,3.

## DISCUSSÃO

Os resultados deste estudo apontam que a necessidade de prótese em idosos brasileiros é elevada, sendo associada a desigualdades socioeconômicas relacionadas à cor da pele, sexo, renda, escolaridade e acesso aos serviços odontológicos. De modo geral, observa-se que não houve mudanças significativas em relação aos cenários de 2003 e 2010^10^ ^,^ ^15^ ^,^ ^17^ ^,^ ^18^. Observa-se, entretanto, que há significativa variação entre os municípios brasileiros, e que a disponibilidade de serviços públicos de saúde bucal não está associada à necessidade de próteses nos idosos.

Esses resultados mostram que a necessidade de prótese em idosos brasileiros é marcada por diferenças socioeconômicas, entre elas a cor da pele. Um estudo que analisou a oferta de próteses dentárias no Brasil revelou que municípios com maior proporção de negros possuem maior iniquidade na oferta de próteses dentárias no SUS^21^. Os achados deste estudo confirmam que idosos pretos e que residem em municípios com maior proporção de negros possuem maior probabilidade de necessitar de próteses. Esses fatores, por sua vez, não foram associados ao uso de prótese.

A variável “cor da pele”, no contexto brasileiro, não representa um marcador biológico, mas sim um marcador social que expressa desigualdades estruturais decorrentes de processos históricos de racialização e racismo estrutural. Estudos confirmam que populações negras enfrentam menor acesso à reabilitação protética e maior prevalência de edentulismo, mesmo após ajuste por renda e escolaridade^9^ ^,^ ^21^. Os achados deste estudo reforçam que os efeitos da raça/cor extrapolam o nível individual, manifestando-se também de forma contextual, uma vez que municípios mais negros apresentam maior prevalência de necessidade de próteses. Isso evidencia um mecanismo de interseccionalidade, no qual raça e condição socioeconômica interagem intensificando vulnerabilidades acumuladas ao longo da vida. Neste estudo, a variável “raça/cor” não foi empregada como proxy socioeconômico, mas incluída no modelo estatístico como um marcador de iniquidade social. Os resultados apontam que pessoas pretas necessitam mais de próteses, embora não tenham sido verificadas diferenças em relação ao uso.

A maior oferta de próteses em municípios com maior número de equipes de saúde bucal na atenção primária e de CEOs, conforme reportado anteriormente^21^, conflita com os resultados deste estudo, que não demonstraram efeitos da cobertura de saúde bucal na APS, nem da presença de CEOs e LRPD nos municípios. Neste estudo, a elevada frequência de indivíduos que residem em municípios com disponibilidade de serviços públicos de saúde bucal pode ter influenciado os resultados. Além disso, é possível que os idosos não procurem o serviço público odontológico para confecção e instalação de próteses dentárias.

Um estudo recente demonstrou que a expansão de equipes de saúde bucal na atenção primária do SUS aconteceu prioritariamente em municípios menos populosos, com menor PIB per capita, na região Nordeste^3^. De certo modo, esses dados ilustram que a PNSB priorizou municípios com maior vulnerabilidade, sendo este indicador também utilizado atualmente para indução/implantação de equipes de saúde bucal. De acordo com os dados deste estudo, não foi possível observar efeito da maior disponibilidade de serviços odontológicos no SUS na necessidade ou no uso de próteses dentárias por idosos brasileiros. Esperava-se que municípios com a presença de CEO e LRPD, por exemplo, pudessem contribuir para menor necessidade e maior uso de prótese, entretanto este efeito não foi observado.

Portanto, sugere-se que os serviços de saúde bucal priorizem indivíduos mais vulneráveis socioeconomicamente. Atualmente não existem protocolos ou diretrizes que orientem o rastreio de usuários com necessidades odontológicas para triagem e atendimento no SUS. Os resultados deste estudo podem contribuir para o desenvolvimento de algoritmos e ferramentas tecnológicas que possam ser implementadas para este fim. Além disso, a expansão de serviços odontológicos do SUS deve considerar locais com maior vulnerabilidade da população, especialmente relacionada à renda, escolaridade e cor da pele.

Em relação ao uso de prótese dentária, verifica-se que as variáveis socioeconômicas individuais também produziram um efeito significativo no desfecho, semelhante ao observado anteriormente na literatura^10^ ^,^ ^15^ ^,^ ^17^ ^,^ ^18^. Idosos com menor renda e menor escolaridade (categorias de referência) apresentaram menor frequência de uso de prótese. Esses grupos também foram os que apresentaram maior necessidade, demonstrando que essa é uma condição proeminente entre idosos brasileiros.

É importante mencionar que o uso de prótese não é um substituto da ausência de dentes. Indivíduos com perdas dentárias podem usar ou não próteses, bem como necessitar ou não de tratamento reabilitador. Portanto, o uso de próteses indica que o indivíduo teve acesso ao tratamento reabilitador. Além disso, estudos nacionais^10^ ^,^ ^15^ ^,^ ^18^ demonstram que uso e necessidade de próteses têm determinantes parcialmente distintos da perda dentária.

A ausência de diferenças em relação ao uso de prótese nas categorias extremas de renda e escolaridade provavelmente se deve ao fato de idosos com maior renda e maior escolaridade possuírem menor prevalência de edentulismo^9^, enquanto aqueles de menor renda e menor escolaridade (categorias de referência) podem enfrentar barreiras de acesso aos serviços odontológicos^13^ ^,^ ^14^, especialmente no setor público, que também contribuiu significativamente para menor frequência de uso.

No presente estudo, os idosos que utilizaram o serviço público odontológico tiveram menor frequência de necessidade e de uso de prótese dentária. Além disso, a disponibilidade de serviços públicos odontológicos não influenciou as frequências de uso e necessidade de próteses. Esses resultados se justificam, provavelmente, pela evidência da baixa utilização de serviços odontológicos por idosos^13^ ^,^ ^14^. Entretanto, observa-se que tanto a utilização de serviços odontológicos quanto o uso e a necessidade de prótese são marcados por iniquidades socioeconômicas. Portanto, são necessárias medidas para reduzir essas desigualdades e direcionar as ações de saúde bucal do SUS para a população que mais necessita.

Este estudo possui limitações relacionadas a taxa de não resposta, categorização das variáveis em estudo, característica dos participantes e desequilíbrio produzido pelo delineamento amostral complexo. Em relação à taxa de não resposta - aproximadamente 27% dos dados de renda -, mesmo com a exclusão desses indivíduos da amostra, verificou-se que os modelos estatísticos se apresentaram adequadamente ajustados e robustos para produzir inferências.

No presente estudo, o tipo de prótese dentária não foi considerado na análise do uso e da necessidade em idosos brasileiros. Do ponto de vista organizacional, de disponibilização de recursos para o serviço, os tipos de próteses dentárias produzidas não são diferenciados, mas apenas o quantitativo destas, de forma que os LRPD são incluídos nas faixas produtivas (sem discernir tipos de próteses). Desse modo, a análise dos fatores associados ao uso e à necessidade de prótese em idosos brasileiros não tem qualquer prejuízo.

As análises conduzidas neste estudo incluíram todos os idosos, independentemente da adesão a planos odontológicos privados. Assim, optou-se por incluir todos os participantes, tendo em vista obter um panorama da totalidade da população idosa brasileira. Ao considerar o SUS como um sistema universal, usuários de planos privados também devem ser incluídos na abrangência do serviço público de saúde.

As dicotomizações adotadas neste estudo decorrem das variáveis contextuais, que têm por base indicadores do Ministério da Saúde, a exemplo da cobertura de saúde bucal e presença de CEO, que são utilizadas para orientar políticas públicas. Já no que tange às variáveis individuais, o presente estudo buscou adotar múltiplas categorias, possibilitando a avaliação de existência de gradientes sociais.

Embora os CEOs não sejam necessariamente responsáveis pela oferta de próteses dentárias, esses serviços representam um esforço da gestão local na oferta de atenção especializada. Em muitos locais o atendimento clínico para confecção de próteses é integrado ao CEO, ainda que a efetiva oferta das próteses seja dependente da estrutura dos LRPD. Outrossim, a presença de CEOs pode representar maior estruturação da rede de atenção à saúde bucal, maior qualificação dos fluxos assistenciais e maior probabilidade de encaminhamento para LRPD. Estudos prévios demonstram que municípios com maior densidade de serviços especializados apresentam melhor desempenho geral da rede de saúde bucal. Assim, incluímos essa variável como um indicador de capacidade instalada e organização da rede, o que pode indireta ou diretamente influenciar desfechos de reabilitação.

Além disso, o maior peso amostral de indivíduos residentes em capitais pode estar associado a maior disponibilidade de CEOs e LRPD, e menor cobertura de saúde bucal na APS. É importante considerar, também, que parte significativa desses indivíduos pode não procurar o atendimento odontológico no SUS. Portanto, a influência da disponibilidade de serviços públicos odontológicos deve ser analisada com cautela.

Os resultados desta investigação permitem concluir que variáveis socioeconômicas individuais influenciam significativamente a necessidade e o uso de próteses dentárias em idosos. Entre as variáveis contextuais, apenas a maior proporção de negros dos municípios foi associada a maior necessidade de prótese dentária, revelando que o contexto municipal também pode influenciar os resultados e destacando, inclusive, iniquidades possivelmente relacionadas ao racismo.

Considerando que a necessidade e o uso de próteses em idosos brasileiros permaneceram inalterados entre 2010 e 2023, é factível que a expansão da oferta de serviços odontológicos no SUS não influenciou essas estimativas. Esses achados acendem o alerta para a necessidade de desenvolver estratégias e ferramentas que possam produzir mudanças no uso e na necessidade de próteses na população. É alarmante que 49,6% dos adultos brasileiros (35 a 44 anos) não usem e necessitem de algum tipo de prótese^22^. Portanto, é evidente que a PNSB deve desenvolver estratégias para ampliação da oferta de próteses no SUS. A ausência de ações poderá contribuir para que este cenário permaneça inalterado entre idosos brasileiros nos próximos 10 ou 20 anos.

Sendo assim, o uso e a necessidade de próteses dentárias em idosos brasileiros são marcados por iniquidades socioeconômicas relacionadas a sexo, cor da pele, renda e escolaridade. A utilização de serviços públicos de saúde bucal contribui para maior necessidade e menor uso de prótese dentária em idosos. A proporção de negros nos municípios influenciou a necessidade de próteses, e a disponibilidade de serviços odontológicos no SUS não demonstrou efeito significativo.

Dito isso, faz-se necessária a adoção de técnicas com o objetivo de priorizar a oferta de próteses para pessoas em pior condição de vulnerabilidade, sendo adotadas estratégias que ampliem a eficiência da atenção odontológica prestada por serviços de saúde bucal no SUS.
